# Filler Migration and Granuloma Formation After Gluteal Augmentation with Free-silicone Injections

**DOI:** 10.7759/cureus.3294

**Published:** 2018-09-13

**Authors:** Alexander Leyva, Tri Tran, Andrew T Cibulas, David Warden, Franklin J Danger, Kurt Scherer, Christopher Wasyliw

**Affiliations:** 1 Radiology, Florida Hospital, Winter Park, USA; 2 Undergraduate Studies, University of Central Florida, Orlando, USA; 3 Radiology, Florida Hospital-Orlando, Orlando, USA; 4 Diagnostic Radiology, Florida Hospital-Orlando, Orlando, USA

**Keywords:** free silicone, gluteal augmentation, filler migration, silicone injections, granulomas, liquid silicone, adulterated silicone, silicone migration, granuloma formation

## Abstract

Gluteal augmentation may be performed using a variety of techniques, including implant-based, autologous fat grafting, local flaps, impermanent filler injection, or, as in this case, by way of permanent filler injection with free-silicone. Of these, free-silicone injections carry one of the highest complication rates, specifically regarding migration of the filler material from the native injection site and induction of painful reactive soft tissue changes at the new filler location. A radiologist providing this diagnosis may assist the clinician, who often cannot obtain a history of illicit silicone injection for gluteal augmentation unless the suspicion is raised. Presented here is a case of painful filler migration to the knee with granuloma formation after free-silicone gluteal injection.

## Introduction

Gluteal augmentation has become increasingly popular as a means of enhancing physical appearance. The most popular methods involve implant-based techniques, as well as autologous fat injection. Additional, less common techniques include local fat or muscle flaps, resorbable dermal filler injection using hyaluronic acid gel, and permanent filler injection using free liquid silicone [1, 2]. Free-silicone injection is an attractive option to many patients due to its relative low cost [3, 4]. However, many cases of complication development due to the use of silicone for buttock, and similarly breast, enhancement have been reported. Rates of complications are especially high when adulterated silicone and large volumes are used, which often contains oil-based byproducts along with other often poorly-tolerated impurities [5-8]. Of the possible complications, filler migration with resultant granuloma formation is relatively common and thus important to recognize. Even when pure medical-grade silicone is used, granuloma formation can occur in up to 20% of patients, especially with higher volume injections [9, 10].

## Case presentation

**Figure 1:**
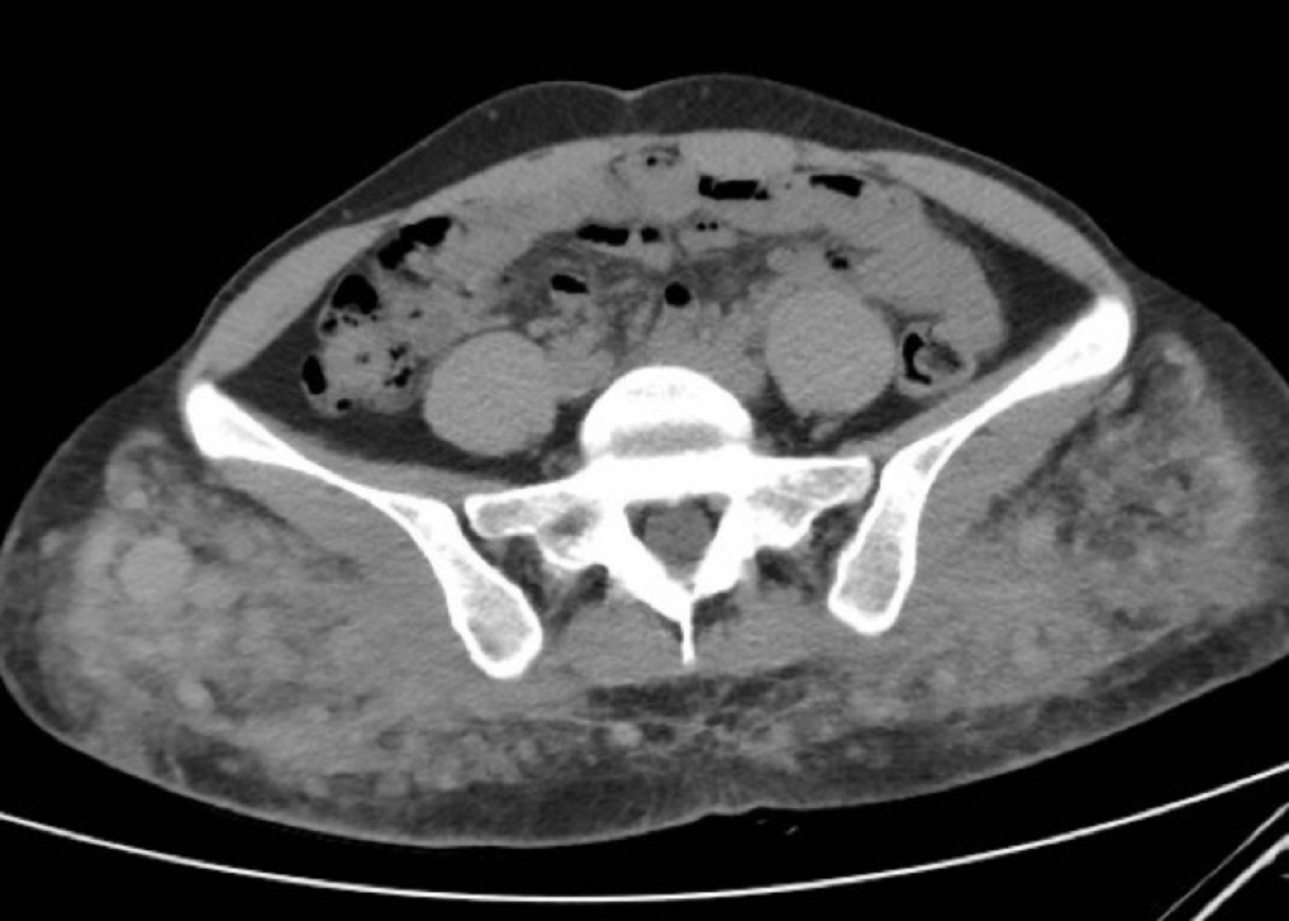
CT Abdomen/Pelvis Axial CT image of the pelvis demonstrates sites of prior subcutaneous gluteal free-silicone injection with associated reactive soft tissue changes, including granuloma formation.

**Figure 2:**
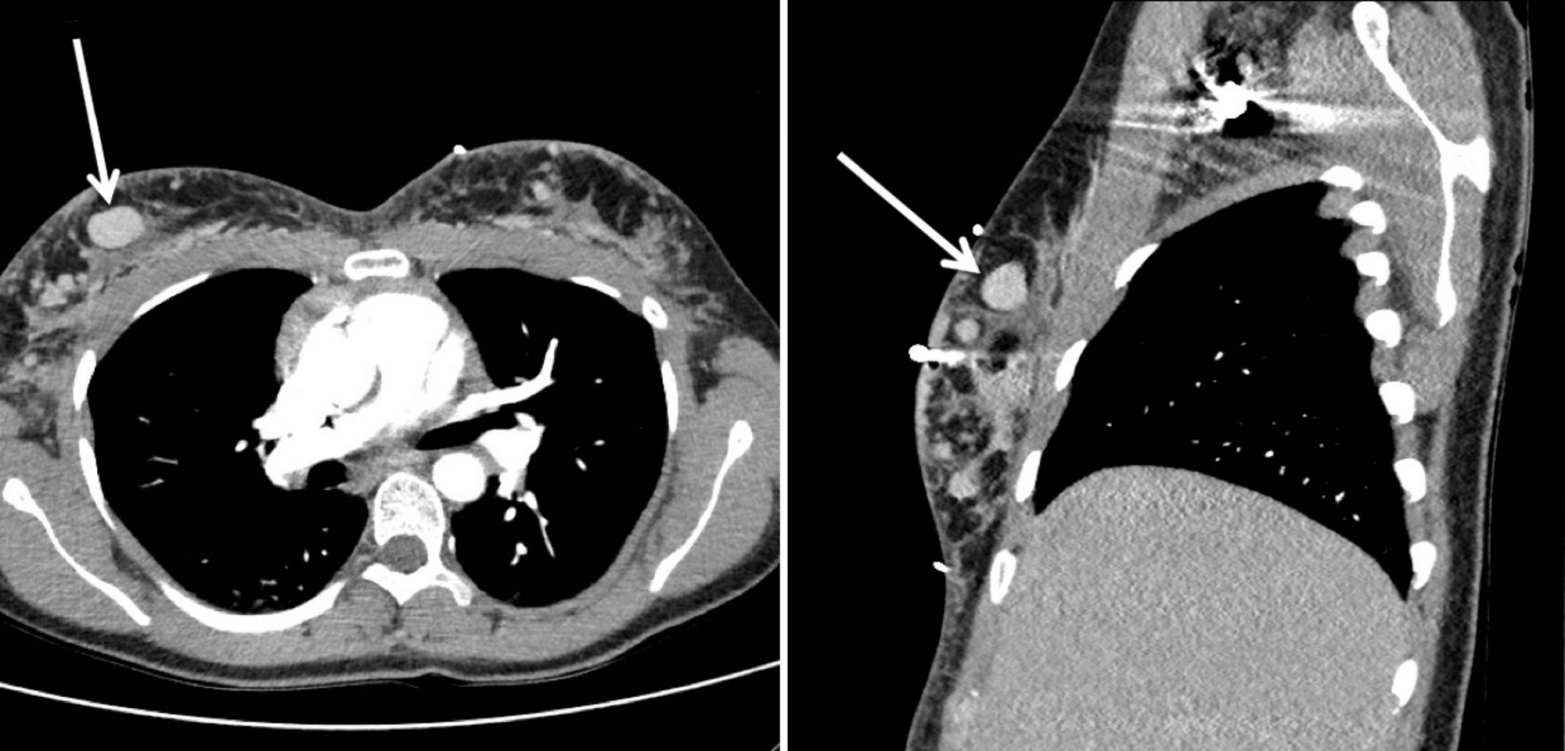
CT Chest Axial* (left)* and sagittal *(right) *contrast-enhanced CT images of the chest demonstrate bilateral areas of prior free-silicone injection with surrounding granulomatous change. The largest globule of free silicone is visualized within the right breast *(arrows)*.

**Figure 3:**
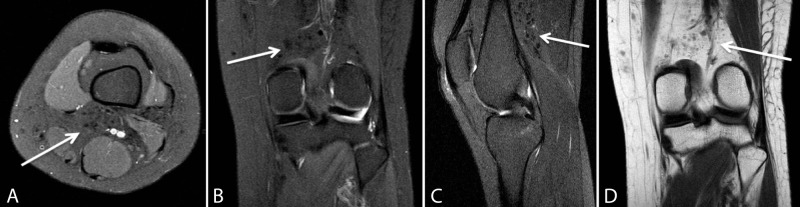
MRI Left Knee Axial proton-density *(A)*, coronal proton-density *(B)*, sagittal T2-weighted *(C)*, and coronal T1-weighted *(D)* MR images of the left knee demonstrate translocated free-silicone and reactive granuloma formation *(arrows) *within the posterior knee.

A 28-year-old woman with a history of uterine malignancy, deep vein thrombosis, and hepatitis B, who had been recently admitted as an inpatient for management of renal calculi presented with left knee pain. A magnetic resonance imaging (MRI) scan of the left knee was subsequently ordered by the patient’s clinician. Prior to prescribing an MRI of the left knee, the patient had undergone a recent computerized tomography (CT) scan of the abdomen and pelvis, which had demonstrated numerous partially calcified granulomas in the gluteal subcutaneous tissues bilaterally (Figure 1). Additionally, a recent chest CT demonstrated similar findings of prior free-silicone injections within the bilateral breasts and surrounding granulomatous change (Figure 2). MRI of the left knee revealed scattered, small circumscribed areas of signal abnormality in the posterior distal thigh, as well as within the subcutaneous tissues and fascia of the popliteal fossa. These round structures were low in signal on proton density, T1, and T2-weighted sequences (Figure 3).

## Discussion

Silicone is a substance with a controversial history. It belongs to a family of synthetic polymers comprised of the element silicon, of which the liquid form was first used for breast enhancement in the United States in 1963 [4, 9]. Due to increasing rates of associated complications, Nevada became the first state to criminalize the use of free-silicone in 1975 [4]. Almost two decades later, in 1992, after implant-based methods of breast augmentation had been developed, the U.S. Food and Drug Administration (FDA) temporarily banned the use of silicone breast implants to conduct studies on its safety, eventually lifting the ban in 2006 [10]. Today, free-silicone injection is only approved for ophthalmic use in the setting of retinal detachment [4].

Although safety remains a topic of debate, silicone-related procedures continue to be performed routinely, and often illegally, in many countries for cosmetic purposes [4, 10]. The patient presented here had obtained illicit free-silicone injections for breast and buttock augmentation. Given the illicit nature of this procedure, the patient initially failed to disclose this information. In cases with such limited available history, it may be difficult for clinicians to effectively diagnose conditions leading to painful granuloma formation. Imaging findings, such as those previously discussed involving various sites with multi-focal reactive soft tissue changes and granuloma formation, offer clues to the final diagnosis. As a result, the practicing radiologist may assist the clinician in considering this diagnosis by evaluating for the typical CT and MR imaging features previously outlined.

The final diagnosis for this case included granuloma formation in the posterior thigh and popliteal fossa with caudal migration of free-silicone to the left knee. Findings on provided MR images, however, are not specific for the diagnosis. In conjunction with the aforementioned CT imaging features of the pelvis and chest, demonstrating probable injection sites with surrounding reactive soft tissue change, the final radiologic diagnosis may be suggested.

## Conclusions

This case demonstrates complications from an unusual form of gluteal and breast augmentation by free-silicone injection. Since patients do not typically disclose undergoing illicit procedures, the practicing radiologist may assist the clinician in proposing the diagnosis when the appropriate imaging findings are present.
